# The effects of MOTILIPERM on cisplatin induced testicular toxicity in Sprague–Dawley rats

**DOI:** 10.1186/s12935-015-0274-1

**Published:** 2015-12-18

**Authors:** Kiran Kumar Soni, Li Tao Zhang, Jae Hyung You, Sung Won Lee, Chul Young Kim, Wan Shou Cui, Han Jung Chae, Hye Kyung Kim, Jong Kwan Park

**Affiliations:** Department of Urology, Institute for Medical Sciences, Chonbuk National University of Medical School, Jeonju, 561-712 Republic of Korea; Biomedical Research Institute and Clinical Trial Center for Medical Devices of Chonbuk National University Hospital, Jeonju, 561-712 Republic of Korea; Department of Urology, Samsung Medical Center, Samsung Biomedical Research Institute, Sungkyunkwan University Medical School, Seoul, Republic of Korea; College of Pharmacy, Hangyang University, Ansan, 426-791 Republic of Korea; Andrology Center, Peking University First Hospital, Beijing, 100034 China; Department of Pharmacology, Chonbuk University Medical School, Jeonju, Republic of Korea

**Keywords:** Cispaltin (CIS), MOTILIPERM, Spermatogenic cell denity, Steroidogenic acute regulatory (StAR) protein, Glucose-regulated protein-78 (GRP-78), Phosphorylated inositol-requiring transmembrane kinase/endoribonuclease 1 (IRE1), Phosphorylated c-jun-N-terminal kinase (p-JNK)

## Abstract

**Background:**

Cisplatin causes male infertility but the exact mechanism have not been clarified, yet. MOTILIPERM has been implicated in alleviation of infertility in Sprague–Dawley rats caused by cisplatin. We evaluated recovery effect of MOTILIPERM on cisplatin (CIS)-induced testicular toxicity in Sprague–Dawley rats.

**Methods:**

Five groups were included. The groups are control (CTR), CTR + MOTILIPERM 200 mg/kg/day per oral, CIS 10 mg/kg i.v., CIS 10 mg/kg + MOTILIPERM 100 mg/kg/day, CIS 10 mg/kg + MOTILIPERM 200 mg/kg/day. CIS 10 mg/kg i.v. single dose was given before 100 mg/kg, or 200 mg/kg MOTILIPERM per oral daily for 28 days. Body and genital organs weight, epididymis sperm count, sperm motility, sperm apoptosis, testosterone level, MDA of testis tissue, spermatogenic cell density, and Johnsen’s score were evaluated. Steroidogenic acute regulatory (StAR) protein, and Glucose-regulated protein-78 (GRP-78), phosphorylated Inositol-Requiring Transmembrane Kinase/Endoribonuclease 1 (IRE1) and phosphorylated c-jun-N-terminal kinase (p-JNK) were quantitated by western blot to show its signaling pathway.

**Results:**

The body weight was decreased significantly in CIS 10 mg/kg, CIS 10 mg/kg + MOTILIPERM 100 mg/kg/day, CIS 10 mg/kg + MOTILIPERM 200 mg/kg/day compared with CTR (*p* < 0.001) however, it was increased in CIS 10 mg/kg + MOTILIPERM 100 mg/kg/day, CIS 10 mg/kg + MOTILIPERM 200 mg/kg/day compared with CIS 10 mg/kg. The decreased weight of epididymis and prostate were increased significantly in CIS 10 mg/kg + MOTILIPERM 100 mg/kg/day compared with CIS 10 mg/kg. Sperm count, sperm motility, sperm apoptosis, MDA of testis tissue, spermatogenic cell density, Johnsen’s score, and total testosterone were also significantly improved by MOTILIPERM treatment. The levels of decreased StAR protein was significantly improved by MOTILIPERM administration, increased GRP-78 protein p-IRE1and p-JNK was also significantly decreased with MOTILIPREM treatment.

**Conclusion:**

The MOTILIPERM could be an effective medicine to reduce the toxic effect caused ER stress by CIS in the testis.

## Background

Cis-diamminedichloroplatinum (II) (cisplatin or cisplatinum, CIS), an antineoplastic drug made in the end of the 19th century, around the peculiar atomic configuration of platinum and was described by the Italian chemist Michele Peyrone [1, 2]. CIS has been used worldwide for treatment of other solid neoplasms, including head and neck, lung, colorectal, hematologic, and ovarian cancers [3, 5]. In 1978 the US Food and Drug Administration (FDA) approved the use of CIS for use in testicular and bladder cancer patients [4].

Effects of CIS treatment on testicular function have been noted in human [6] and other animal models [7, 8]. Animals administered CIS develop severe testicular damage characterized by germ cell apoptosis, Leydig cell dysfunction and testicular steroidogenic disorder leading to infertility. Spermatogenesis is affected by CIS by inhibiting nucleic acid synthesis of germ cells [10]. CIS also inhibit testosterone production by damage of Leydig cells [11].

CIS forms covalent adduct with the cellular DNA molecules and terminate the vital processes like replication and transcription and induce apoptosis [12]. The molecular mechanism by which CIS causes reproductive toxicity and germ-cell apoptosis remains to be elucidated [13, 14].

Alternative methods therefore different herbal medicines like *Zingiber officinale and Hibiscus sabdariffa,Curcuma longa, Ginkgo biloba* or other agents like melatonin, amifostine are used to improve the infertility caused by CIS [8, 14, 25, 27, 30].

*Cuscuta chinensis*, a Chinese Dodder is a parasitic plant. It is commonly used in traditional medicine. It is often used to improve sexual potency [16]. Total flavones from *Cuscutachinensis* seeds can increase the testosterone level in the testicle [17]. *Allium cepa* has antioxidative and androgenic effects in rats that promote spermatogenesis cycle [19, 21, 22]. *Morinda officinalis* is also used to improve the sperm motility [18]. Usually one or the mixture of two herbal medicines are used to treat the testicular toxicity, but we have selected three different herbal medicines to improve the male fertility who are treated with CIS. We used this mixture of three to clarify whether they can improve the CIS inducing male infertility.

## Methods

### Animals, chemicals, experiment protocol

The study was approved by the Ethical Committee of Chonbuk National University followed Basel Declaration. Sexually mature male SD rats weighing 250–300 gm and 9–10 weeks of age were used. The rats were randomly divided into five groups containing 10 rats each, except for the control group with 6 rats. The rats were fed standard rat chow prepared by Feedlab (Guri, Gyeonggi, South Korea) and had access to water. They were maintained in the animal facility under constant environmental conditions (room temperature 20 ± 2 °C, relative humidity 50 ± 10 %, and 12-hour light–dark cycle).

CIS was purchased from Wako Pure Chemical Industries, Ltd. (Doshomachi, Osaka, Japan). The five groups were control (CTR) group (*n* = 6), CTR + MOTILIPERM 200 mg/kg per oral (p.o.) (CTR + M 200) group (*n* = 9), CIS 10 mg/kg intravenous (i.v.) (CIS) group (*n* = 8), CIS 10 mg/kg i.v. + MOTILIPERM 100 mg/kg/day p.o. group (CIS + M 100) group (*n* = 10), and CIS 10 mg/kg i.v. + MOTILIPERM 200 mg/kg/day p.o. (CIS + M 200) group (*n* = 7). The remaining rats died as we had taken 10 rats in each group, except the control group. All rats were sacrificed after 28 days. CIS 10 mg/kg body weight mixed in normal saline was given i.v. by the tail vein; this group was sacrificed 7 days after the CIS injection, since we have previously demonstrated the high mortality of rats treated with CIS for more than 7 days. The recovery group that received the CIS was given MOTILIPERM (100 or/and 200) mg/kg/day p.o. for 28 days beginning the day following CIS administration. Doses of MOTILIPERM were selected based on previous experiments in our laboratory. We used a 28-day MOTILIPERM treatment period because the normal spermatogenesis period of rats is 12.9 days [9].

### Plant material

MOTILIPERM is currently under development by the Dong-A Pharmaceutical Company (Kyoungi, South Korea) for the treatment of infertility. It was mixed in normal saline. It included *Morinda officinalis*, *Cuscuta chinensis* and *Allium cepa* (100 or 200 mg/kg/day) was given orally for 28 days.

### Extraction and fractionation of MOTILIPERM

**Fig. 1:**
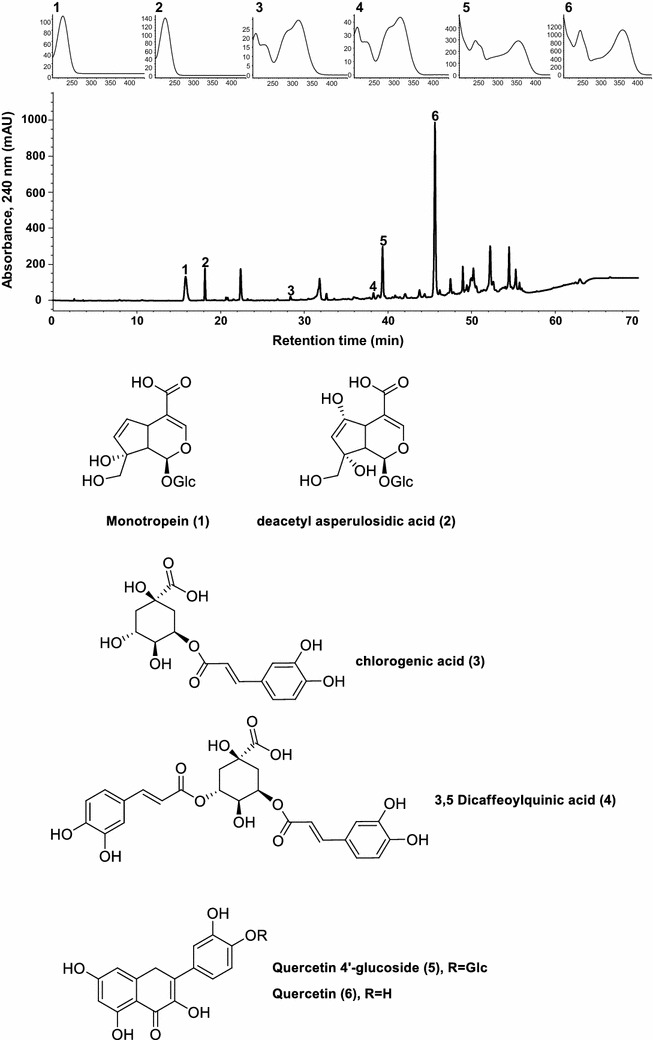
HPLC chromatogram of MOTILIPERM and ultraviolet spectra of major marker components from each herbal ingredients. Monotropein (*1*) and deacetyl asperulosidic acid (*2*) from the *Morinda officinalis*; Chlorogenic acid (*3*) and 3,5-dicaffeoylquinic acid (*4*) from the *Cuscuta japonica*; quercetin 4′-glcoside (*5*) and quercetin (*6*) from the *Allium cepa*. Each peak of MOTILIPERM in the HPLC chromatogram was identified by comparison with the retention times and UV spectra of standard compounds

MOTILIPERM was prepared the mixtures of three medicinal herbs. Each herb was ground and extracted three times with ethanol under reflux for 3 h at 70–80 °C. The combined filtrate was concentrated by rotary evaporator and freeze dried, and stored at −20 °C until required. The quality of MOTILIPERM was confirmed by its principal constituents using high-performance liquid chromatography (HPLC). Twenty milligrams of MOTILIPERM was dissolved in 30 mLmethanol and filtered through a 0.45 μM membrane filter. Ten microliters of the filtrate was injected into the HPLC system for analysis. Each peak of MOTILIPERM in the HPLC profile was identified by comparison with the retention times and UV spectra of standard compounds. An Agilent 1200 HPLC system with an INNO column C18 (4.6 × 250 mm, YoungJin Biochrom, Seoul, South Korea) was used at a flow-rate of 1.0 mL/min at 40 °C. The mobile phase consisted of acetonitrile containing 0.1 % trifluoroacetic acid (solvent A) and water containing 0.1 % trifluoroacetic acid (solvent B). A linear gradient system consisted of 0–0 % A for 0–10 min, 0–30 % A for 10–40 min, 30–50 % A for 40–50 min, 50–100 % A for 50–60 min and 100 % A for 60–70 min. The chromatographic profile of the effluents was recorded at 240 and 254 nm with a spectrum ranging from 210 to 450 nm. The HPLC profile of MOTILIPERM and its identified compounds are shown in Fig. 1.

### Samples collection

The rats were sacrificed using a mixture of Ketamin (100 mg/mL) and 2 % rumpun (20 mg/mL).

### Sperm count and motility

The epididymis was minced and suspended in normal saline at 37 °C for 5 min. The sperm suspension was placed on a sperm-counting chamber (SEFI-Medical Instruments, Haifa, Israel) with a pipettor. The chamber was warmed to 37 °C before sperm counts and motility were assessed. The total sperm count was calculated using two or three drops of the specimen to increase the reliability of count determination. Sperm heads were counted in 10 squares. The recorded sperm count represents the concentration of sperm as millions of sperm per ml. The average value was reported. The sperm were counted using the 20 X magnification objective on the light microscope.

### Sperm apoptosis

The number of apoptotic sperms was analyzed by flow cytometry using a Gallios™ device (Beckman Coulter, Brea, CA, USA). Propidium iodide was purchased from BD Pharmigen (San Diego, CA, USA). Five microlitre of semen sample was taken in an Eppendorf tube, 100 µL binding buffer was added, 5 µL propidium iodide was added, and the mixture was incubated at room temperature in dark for 20 min. Binding buffer (400 µL) was added and apoptosis was measured within an hour.

### Biochemistry

Blood was collected from rats’ vena cava. For testosterone estimation, 10 µL heparin was added in 1 mL blood and was centrifuged at 3500*g* for 10 min. Plasma was transferred to a 5 mL tube and sealed with the paraffin film. All the samples were sent to the hospital laboratory.

### Malondialdehyde (MDA)

Malondialdehyde is a marker for oxidative stress. Homogenized testis tissue was used for MDA analysis. MDA analysis is based on its reaction with thiobarbituric acid to form a pink complex with absorption maximum at 535 nm [13].

### Histology

For histological estimations, small pieces of testis were fixed in the formalin and stained with hematoxylin and eosin. Sections were examined by light microscopy for spermatogenic cell density measurements. Spermatogenic cell density was determined by measuring the thickness of the germinal cell layer and the diameter of the seminiferous tubules.

Seminiferous tubules were graded by Johnsen’s scoring. In this system of classification, seminiferous tubules are assessed according to the presence of spermatogenic cells and each is given a score from 1 to 10. Complete spermatogenesis with many spermatozoa present is evaluated as score 10. The detailed score histological criteria [28] are: Score 1: No seminiferous epithelium; Score 2: No germinal cells, Sertoli cells only; Score 3: Spermatogonia only; Score 4: No spermatozoa or spermatids, few spermatocytes; Score 5: No spermatozoa or spermatids, many spermatocytes; Score 6: No spermatozoa, no late spermatids, few early spermatids; Score 7: No spermatozoa, no late spermatids, many early spermatids; Score 8: Less than five spermatozoa per tubule, few late spermatids; Score 9: Slightly impaired spermatogenesis, many late spermatids, disorganized epithelium.; and Score 10: Full spermatogenesis.

### Western blot

Glucose-regulated protein-78 (GRP-78), phosphorylated Inositol-Requiring Transmembrane Kinase/Endoribonuclease 1 (IRE1), total IRE1, phosphorylated c-jun-N-terminal kinase (p-JNK) and total JNK measurements in testis tissue were conducted with obtained tissue that had been washed with cold phosphate buffered saline (PBS). Lysis buffer with protease inhibitor was added to tissue, cordless motor pellet pestles was used to pestle and centrifuge at 12,000 rpm for 30 min at 4 °C.

For StAR protein, crude mitochondrial preparation was acquired as previously described [29]. Testis tissue was thoroughly washed in 2 mL of sucrose buffer (0.25 M sucrose, 10 mM tris, 0.1 mM EDTA and pH 7.4) and the homogenate centrifuged at 600 rpm for 15 min. The cloudy supernatant, containing the mitochondria was removed and transferred to another tube which was then centrifuged at 12,000 rpm for 15 min. The resulting mitochondrial pellet was washed in sucrose buffer and recentrifuged at 12,000 rpm for 15 min. The pellet was again re-suspended in sucrose buffer and the protein content determined by the method of Bradford for both the lysates.

The samples were run on 10 % sodium dodecyl sulfate (SDS) gel, transferred on polyvinylidene fluoride (PVDF) membrane by trans-blot^®^ SD semi-dry electrophoretic transfer cell (Bio-Rad, Hercules, CA, USA). After transfer, the membrane was blocked by 10 % Bovine serum albumin (BSA) for an hour and incubated with phosphorylated antibodies p-IRE1(Abcam Cambridge, MA USA), p-JNK (Santa Cruz Biotechnology, Dallas, TX, USA) but non-fat 10 % milk was used for non-phosphorylated antibodies GRP-78, IRE1, and JNK (Santa Cruz Biotechnology, Dallas, TX, USA) for testis tissue. StAR protein (Cell Signaling Technology, Beverly, MA, USA) for mitochondrial extracts with a 1:1000 dilution overnight at 4 °C. The membrane was washed with Tris buffered saline Tween (TBST) three times prior to the addition of 1:5000 dilution of secondary antibody for 1 h. The membrane was washed three times with TBST and processed using enhanced chemiluminescence substrate.

### Statistical analysis

Data are expressed as mean ± SD. The statistical analysis was carried out using SigmaPlot 12.0 (Systat Software, San Jose, CA, USA). *P* < 0.001 was considered statistically significant.

## Results

### Effect of weights of body, testis, epididymis, and prostate

The weights of body in CIS, CIS + M 100, and CIS + M 200 groups were significantly decreased compared with the CTR group. Body weight significantly increased in CIS + M 100, and CIS + M 200 groups compared with CIS group, and in CIS + M 100 compared with CTR + M 200 group. Testis weight had no statistical significance. Epididymis weight in CIS group was significantly decreased compared with CTR group, and in CIS + M 100 compared with CTR + M 200 and CIS groups. There was a significant decrease in prostate weight in CIS, CIS + M 100, and CIS + M 200 groups compared with CTR group, and in CIS + M 200 group compared with CIS group (Table 1).

**Table 1:** The effect of the MOTILIPERM on body, testicular, epididymis and prostate weights

	CTR	CTR + M 200	CIS groups		
			CIS	CIS + M 100	CIS + M 200
Body Wt (gm)	419 ± 37.22	349.6 ± 13.79	338 ± 16.19^**^	386.3 ± 16.15^*,††,##^	358.43 ± 17.82^*,#^
Testis Wt (gm)	2.05 ± 0.13	1.99 ± 0.10	1.95 ± .014	1.97 ± 0.13	1.96 ± .014
Epididymis Wt (gm)	0.70 ± 0.02	0.60 ± 0.05	0.59 ± 0.05^**^	0.66 ± 0.05^†,#^	0.65 ± 0.06
Prostate Wt (gm)	1.48 ± 0.23	1.14 ± 0.18	1.18 ± 0.19^*^	1.1 ± 0.15^*^	0.97 ± 0.16^**,#^

Data are presented in mean ± SD

*Wt* weight; *CTR* control; *CTR* *+* *M 200* CTR + MOTILIPERM 200 mg/kg/day; *CIS* cisplatin 10 mg/kg intravenous (i.v.); *CIS* *+* *M 100* CIS 10 mg/kg, i.v. + MOTILIPERM 100 mg/kg/day; *CIS* *+* *M 200* CIS 10 mg/kg, i.v. + MOTILIPERM 200 mg/kg/day. *M* MOTILIPERM

^*^ *p* < 0.05 vs CTR group, ^†^ *p* < 0.05 vs CTR + M 200 group, ^#^ *p* < 0.05 vs CIS group, ^**^ *p* < 0.001 vs CTR group, ^††^ *p* < 0.001 vs CTR M 200 group 2, ^##^ *p* < 0.001 vs CIS group

### Effect on sperm count

The sperm count in CIS group was significantly decreased compared with CTR + M 200 group (Table 2).

**Table 2:** The effect of the MOTILIPERM on epididymis sperm count, motility and apoptosis

	CTR	CTR + M 200	CIS groups		
			CIS	CIS + M 100	CIS + M 200
Sperm count (10^6^)	25.17 ± 5.95	28.83 ± 6.10	22.06 ± 8.78^†^	25.40 ± 4.65	28.21 ± 4.46
Sperm motility (%)	33.29 ± 19.01	29.13 ± 15.83	17.56 ± 15.16^*^	17.87 ± 8.47^*,†^	19.86 ± 12.91^*^
Sperm apoptosis (%)	17.38 ± 1.85	8.03 ± 2.96	24.68 ± 4.22^*,††^	19.97 ± 4.68^††,#^	19.90 ± 3.56^††,#^

Data are presented in mean ± SD

*CTR* control; *CTR* *+* *M 200* CTR + MOTILIPERM 200 mg/kg/day; *CIS* cisplatin 10 mg/kg intravenous (i.v.); *CIS* *+* *M 100* CIS 10 mg/kg, i.v. + MOTILIPERM 100 mg/kg/day; *CIS* *+* *M 200* CIS 10 mg/kg, i.v. + MOTILIPERM 200 mg/kg/day. *M* MOTILIPERM

^*^ *p* < 0.05 vs CTR group, ^†^ *p* < 0.05 vs CTR + M 200 group 2, ^#^ *p* < 0.05 vs CIS group, ^††^ *p* < 0.001 vs CTR + M 200 group

### Effect on sperm motility

There was significant decrease sperm motility in CIS, CIS + M 100, and CIS + M 200 groups compared with CTR group, and in CIS + M 100 group compared with CTR + M 200 group (Table 2).

### Effect on sperm apoptosis

There was significant increase apoptosis in CIS group compared with CTR group, and in CIS, CIS + M 100, and CIS + M 200 groups compared with CTR + M 200 group. There was significantly decreased apoptosis in CIS + M 100 and CIS + M 200 groups compared with CIS group (Table 2).

### Effect on MDA

**Fig. 2:**
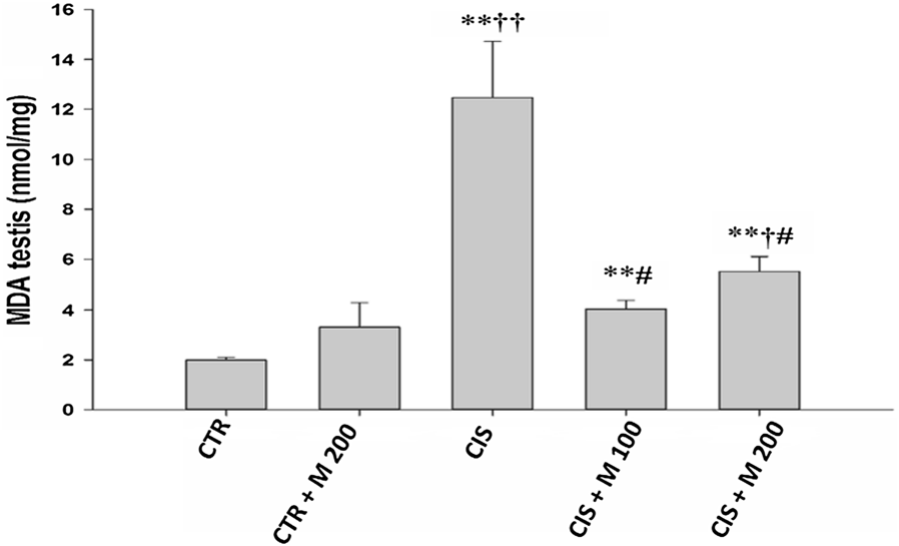
Evaluation of MDA for each group. Data are presented in mean ± SD. ^†^ *p* < 0.05 vs CTR + M 200 group, ^#^ *p* < 0.05 vs CIS group, ^**^ *p* < 0.001 vs CTR group, ^††^ *p* < 0.001 vs CTR + M 200 group

There was significant increase in MDA level in CIS, CIS + M 100 and CIS + M 200 groups compared with CTR and CTR + M 200 groups. There was also significant decrease of MDA level in CIS + M 100 and CIS + M 200 group compared with CIS group (Fig. 2).

### Effect on plasma testosterone and StAR protein

**Fig. 3:**
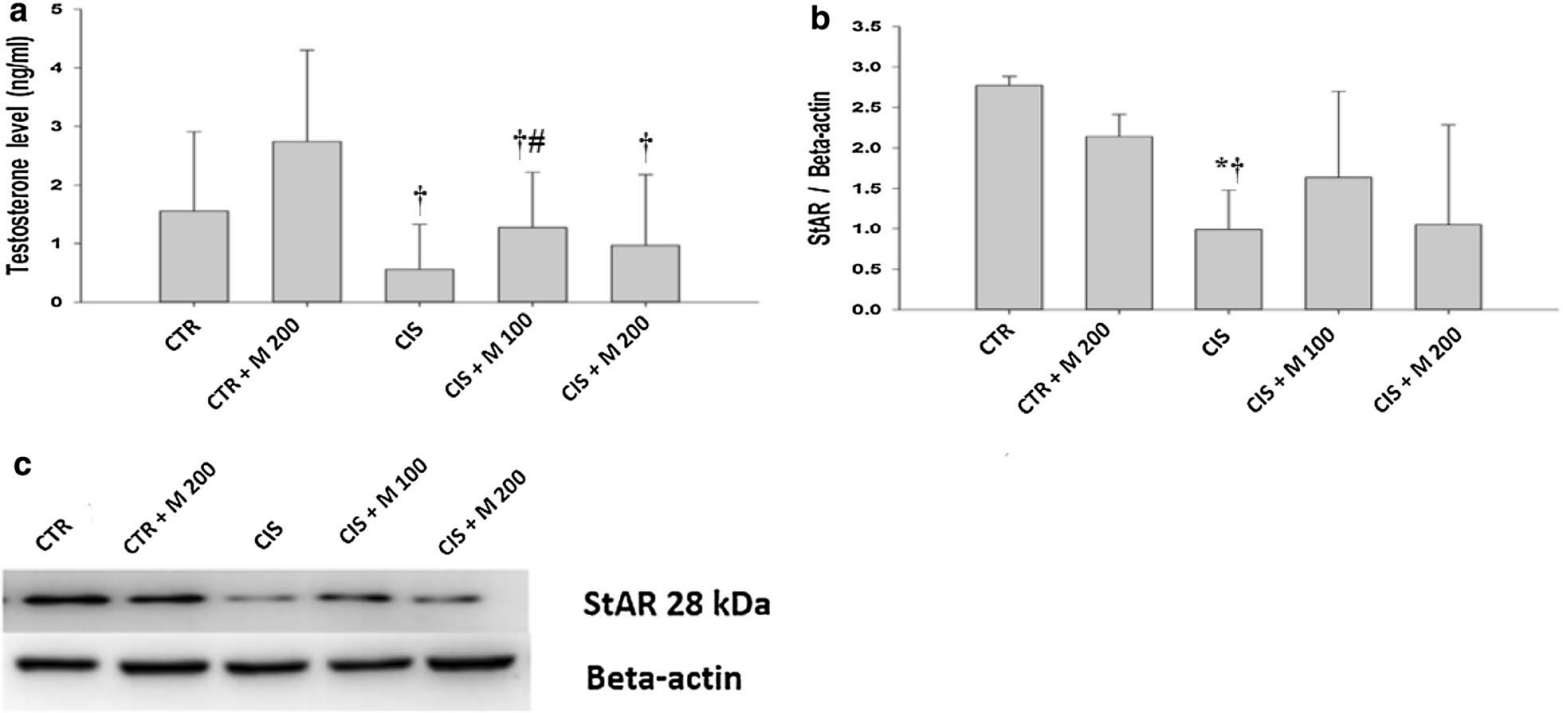
Effects of cisplatin (CIS) and MOTILIPERM on total testosterone and StAR protein by Western blot. **a** Total testosterone level. **b** StAR protein. **c** Western blotting of StAR protein for each group. Data are presented in mean ± SD. *CTR* control; *CTR* *+* *M 200* CTR + MOTILIPERM 200 mg/kg/day; *CIS* cisplatin 10 mg/kg i intravenous (i.v.); *CIS* *+* *M 100* CIS 10 mg/kg, i.v. + MOTILIPERM 100 mg/kg/day; *CIS* *+* *M 200* CIS 10 mg/kg, i.v. + MOTILIPERM 200 mg/kg/day p.o. *M* MOTILIPERM. ^*^ *p* < 0.05 vs CTR group, ^†^ *p* < 0.05 vs CTR + M 200 group, ^#^ *p* < 0.05 vs CIS group

There was a significant decrease of testosterone level in CIS, CIS + M 100, and CIS + M 200 groups compared with CTR + M 200 group (Fig. 3a; Table 3). There was significant increase of testosterone in CIS + M 100 group compared with CIS group. Significantly decreased StAR protein level was evident in CIS group compared with CTR and CTR + M 200 groups. There were no significant changes in CIS + M 100 and CIS + M 200 groups compared with CIS group (Fig. 3b, c).

**Table 3:** The effect of the MOTILIPERM on testosterone assay and spermatogenic cell density

	CTR	CTR + M 200	CIS groups		
			CIS	CIS + M 100	CIS + M 200
Testosterone (ng/ml)	1.56 ± 1.35	2.74 ± 1.56	0.56 ± 0.78^†^	1.27 ± 0.95^†,#^	0.96 ± 1.22^†^
Spermatogenic cell density	0.35 ± 0.07	0.32 ± 0.08	0.18 ± 0.03^*^	0.269 ± 0.028	0.26 ± 0.04

Data are presented in mean ± SD

*CTR* control; *CTR* *+* *M 200* CTR + MOTILIPERM 200 mg/kg/day; *CIS* cisplatin 10 mg/kg intravenous (i.v.); *CIS* *+* *M 100* CIS 10 mg/kg, i.v. + MOTILIPERM 100 mg/kg/day; *CIS* *+* *M 200* CIS 10 mg/kg, i.v. + MOTILIPERM 200 mg/kg/day

^*^ *p* < 0.05 vs CTR group, ^†^ *p* < 0.05 vs CTR + M 200 group, ^#^ *p* < 0.05 vs CIS group

### Histopathological effects

**Fig. 4:**
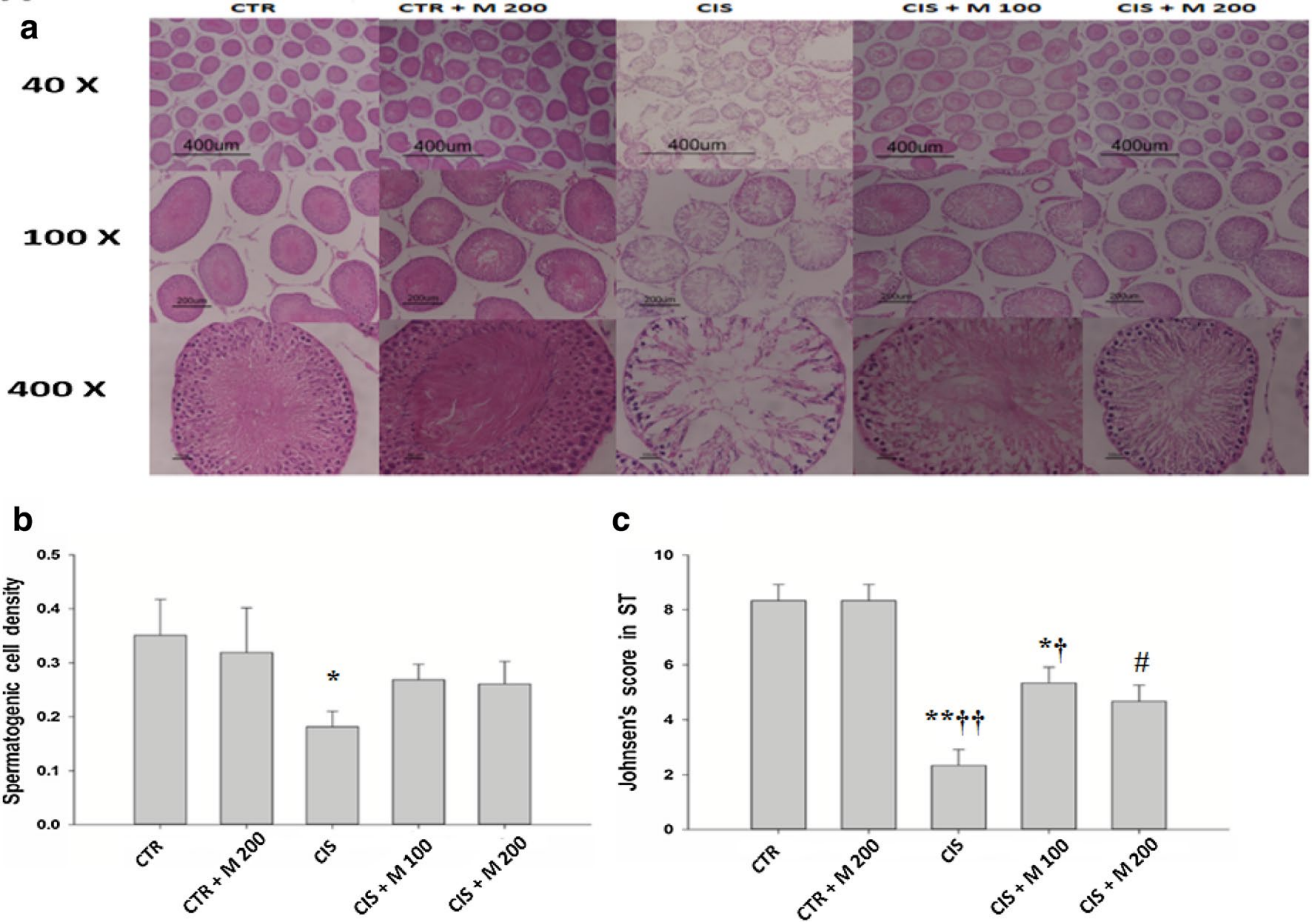
Light microscope evaluations of the testis. **a** Hematoxylin & Eosin (H&E) staining of the testis. **b** Spermatogenic cell density. **c** Johnsen’s score in seminiferous tubules. Data are presented in mean ± SD. *CTR* control; *CTR* *+* *M 200* CTR + MOTILIPERM 200 mg/kg/day; *CIS* cisplatin 10 mg/kg i intravenous (i.v.); *CIS* *+* *M 100* CIS 10 mg/kg, i.v. + MOTILIPERM 100 mg/kg/day; *CIS* *+* *M 200* CIS 10 mg/kg, i.v. + MOTILIPERM 200 mg/kg/day p.o. *M* MOTILIPERM. ^*^ *p* < 0.05 vs CTR group, ^†^ *p* < 0.05 vs CTR + M 200 group, ^#^ *p* < 0.05 vs CIS group, ^**^ *p* < 0.001 vs CTR group, ^††^ *p* < 0.001 vs CTR + M 200 group

Testis tissue showed normal arrangement of the germinal cells, Sertoli cells, and Leydig cells without histopathological lesions in CTR and CTR + M 200 groups. CIS group showed degeneration and disorganization of germinal cells, Sertoli cells, and Leydig cells. CIS + M 100 and CIS + M 200 groups showed the degeneration and disorganization of the testis tissue, but less than in CIS group. There was a significant decrease in the spermatogenic cell density in CIS group compared with CTR group **(**Fig. 4a, b; Table 3). Johnsen’s score was significantly decreased in CIS and CIS + M 100 group compared with CTR and CTR + M 200 groups, and it also was significantly increased in CIS + M 200 group compared with CIS group (Fig. 4c**)**.

### GRP-78, p-IRE1 and p-JNK

**Fig. 5:**
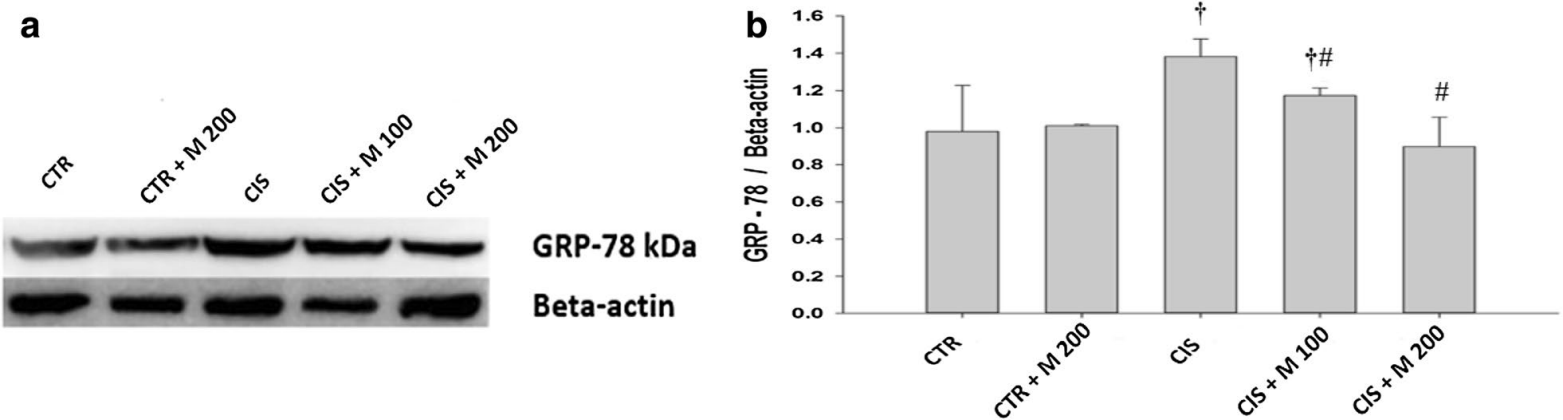
Evaluation of GRP-78. **a** Western blot of testis. **b** Levels of GRP-78 for each group. Data are presented in mean ± SD. *CTR* control; *CTR* *+* *M 200* CTR + MOTILIPERM 200 mg/kg/day; *CIS* cisplatin 10 mg/kg i intravenous (i.v.); *CIS* *+* *M 100* CIS 10 mg/kg, i.v. + MOTILIPERM 100 mg/kg/day; *CIS* *+* *M 200* CIS 10 mg/kg, i.v. + MOTILIPERM 200 mg/kg/day p.o. *M* MOTILIPERM. ^†^ *p* < 0.05 vs CTR + M 200 group, ^#^ *p* < 0.05 vs CIS group

**Fig. 6:**
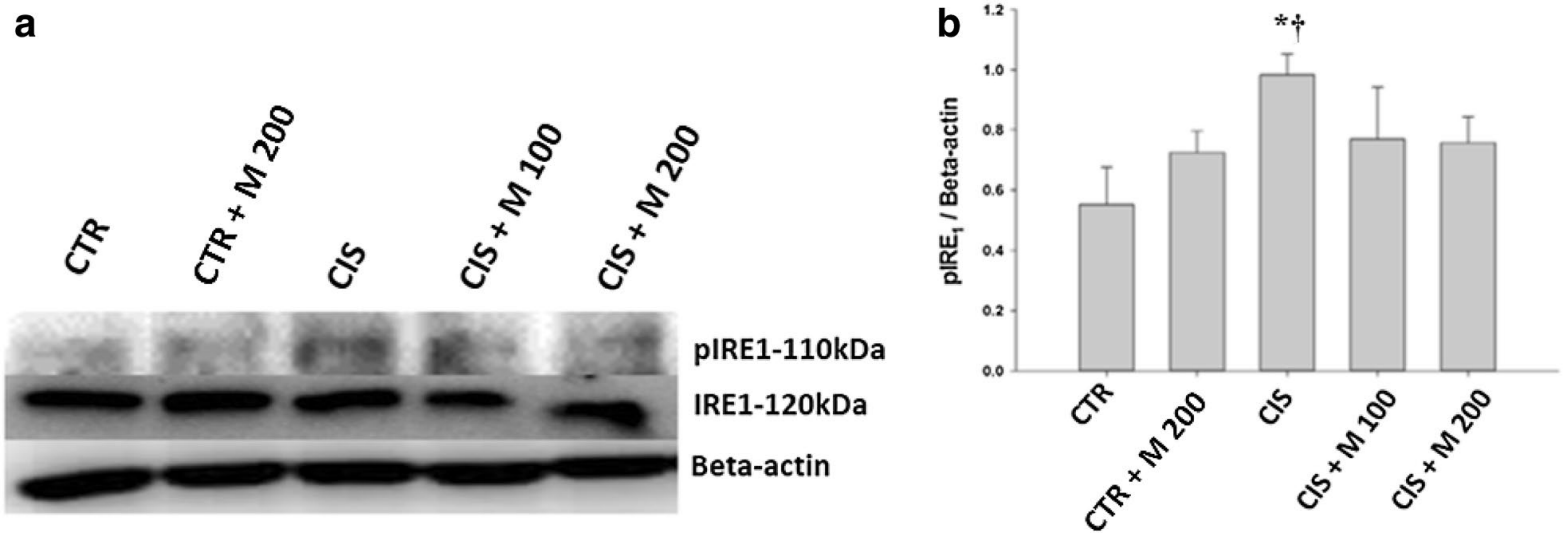
Evaluation of p-IRE1, IRE1, **a** Western blot of testis. **b** Levels of pIRE1 and total IRE1 for each group. Data are presented in mean ± SD. *CTR* control; *CTR* *+* *M 200* CTR + MOTILIPERM 200 mg/kg/day; *CIS* cisplatin 10 mg/kg i intravenous (i.v.); *CIS* *+* *M 100* CIS 10 mg/kg, i.v. + MOTILIPERM 100 mg/kg/day; *CIS* *+* *M 200* CIS 10 mg/kg, i.v. + MOTILIPERM 200 mg/kg/day p.o. *M* MOTILIPERM. ^*^ *p* < 0.005 vs CTR group, ^†^ *p* < 0.05 vs CTR + M 200 group

**Fig. 7:**
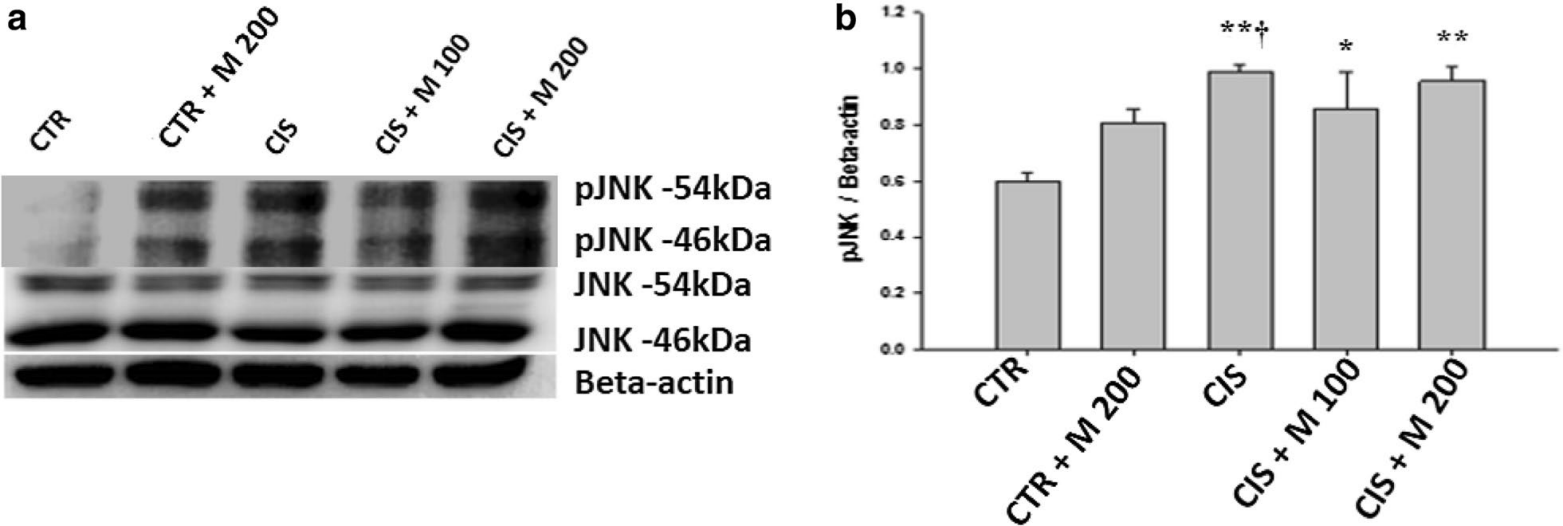
Evaluation of pJNK and JNK **a** Western blot of testis. **b** Levels of pJNK and total JNK for each group. Data are presented in mean ± SD. *CTR* control; *CTR* *+* *M 200* CTR + MOTILIPERM 200 mg/kg/day; *CIS* cisplatin 10 mg/kg i intravenous (i.v.); *CIS* *+* *M 100* CIS 10 mg/kg, i.v. + MOTILIPERM 100 mg/kg/day; *CIS* *+* *M 200* CIS 10 mg/kg, i.v. + MOTILIPERM 200 mg/kg/day p.o. *M* MOTILIPERM.^. *^ *p* < 0.05 vs CTR group, ^†^ *p* < 0.05 vs CTR + M 200 group, ^**^ *p* < 0.001 vs CTR group

Significantly increased GRP-78 was evident in CIS and CIS + M 100 groups compared with CTR + M 200 group. CIS + M 100 and CIS + M 200 groups showed significantly decreased GRP-78 compared with CIS group (Fig. 5a, b). p-IRE1 was significantly increased in CIS group compared to CTR and CTR + M200 groups (Fig. 6a, b). Significantly increased p-JNK was evident in CIS, CIS + M 100, and CIS + M 200 groups compared with CTR group. CIS and group also showed significantly increased compared with CTR + M 200 group (Fig. 7a, b).

## Discussion

CIS is one of the potent anticancer drugs in the chemotherapy treatment; it induces a testicular disintegration, sperm dysfunction, germ-cell apoptosis, and abnormalities in Leydig cells in rats [7, 23]. CIS has significant decrease in the physical weight, reproductive organ weights, plasma testosterone level and spermatozoa compared with untreated control animals. CIS shows impaired fertility along with alterations on the growth and development of the next generations [26].

Thus, the toxic effects of CIS on the germ cells led for further research either to preserve the fertility in men who are undergoing chemotherapy. Semen preservation for future use has increased the chances of pregnancies. But freezing and thawing of semen can reduce the sperm quality [15].

The studies showed the protective effects of herbal plants extracts against CIS due to the presence of antioxidant effects in the herbal medicine [24]. Peng et al. studied about the effects of *Cuscuta chinensis* on human sperm motility in vitro function. The results showed that the motility of sperm significantly improved and the function of sperm membrane became more stable after incubation [18]. In young men antioxidants protect DNA from oxidation and damage and improve the sperm quality [20].

CIS damages testicular tissue and reduces sperm production through increasing oxidative stress and inducing apoptosis and upregulations of nuclear factor kappa-light-chain-enhancer of activated B cells (NF-kB), inducible nitric oxide synthase (iNOS), and cyclooxygenase-2 (COX-2), while *Ginkgo biloba* reduces these oxidative and apoptosis actions of CIS in testis [25]. CIS activate mitogen-activated protein kinases (MAPK), nuclear factor-kappa NF-kB and nitric oxide synthase iNOS expression that has role in pathogenesis in rat testis induced by CIS is blocked by antioxidant such as *Curcuma longa* [30].

*Zingiber officinale and Hibiscus sabdariffa* have also been reported to prevent CIS related damage in testicular tissue and sperm cells by its antioxidant and anti-inflammatory agents [8]. Besides herbal medicines melatonin decrease malondialdehyde (MDA) and increase glutathione (GSH) levels in several pathological conditions like CIS toxicity [14]. Amifostine partially protect the rat seminiferous epithelium against CIS toxicity [27].

**Fig. 8:**
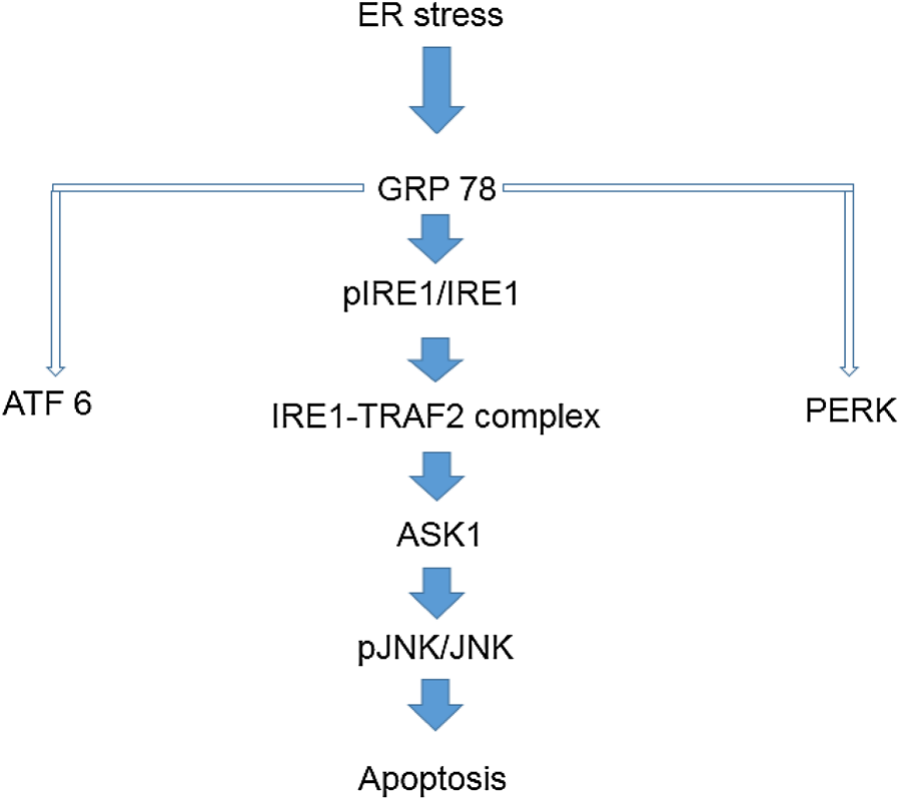
Schematic view of endoplasmic reticulum (ER) stress signaling pathway explained. *GRP78* Glucose-regulated protein-78 (GRP-78), *pIRE1* phosphorylated inositol-requiring transmembrane kinase/endoribonuclease1; *IRE1* inositol-requiring transmembrane kinase/endoribonuclease1; *ATF6* activating transcription factor-6; *TRAF2* tumor necrosis factor receptor associated factor2; *PERK* protein kinase RNA-like endoplasmic reticulum kinase; *ASK1* apoptosis signal-regulating kinase; *pJNK* phosphorylated c-jun-N-terminal kinase; *JNK* c-jun-N-terminal kinase

There are three signaling pathways from ER Stress sensors [31], they are Activating Transcription Factor-6 (ATF6), Protein kinase RNA-like endoplasmic reticulum kinase (PERK) and Inositol-Requiring Transmembrane Kinase/Endoribonuclease 1 (IRE1). Prolonged ER stress activates IRE1 and it may interacts with tumor necrosis factor receptor associated factor 2 (TRAF2) [32]. The IRE1-TRAF2 complex may activate (Apoptosis signal-regulating kinase) ASK1, also known as MAP kinase kinase, leading to activation of JNK pathway. ER stress-induced activation of the ASK1-JNK pathway may trigger apoptosis (Fig. 8) [33].

In our study we have designed to show CIS administration caused testicular toxicity within a week, which recovers by administration of MOTILIPERM orally in 28 days. MOTILIPERM of different doses offers recovery of apoptotic changes against the CIS. This recovery effect was seen in the sperm cell by the flow cytometry where apoptosis was more in CIS group than MOTILIPERM treated group just after CIS administration. It was further accompanied with the restoration of body weight where the testis weight and epididymis weight both were restored but in some part, CIS + M 100 group showed better results than the CIS + M 200 group. The exact mechanism of it is not so clear. Sperm count and sperm motility of the epididymis were also seen to increase compared to the CIS group. MDA level was increased in CIS treated groups.

Total testosterone level was also increased in the recovery group where MOTILIPERM is given just after CIS administration. CIS cause damage of the Leydig cells [11], so the testosterone production is hampered. The StAR protein in the testis tissue showed decreased level in CIS group and recovery groups compared with the CTR, M-200 group in western blot results.

GRP-78, p-IRE1, and p-JNK proteins were increased in CIS, CIS + M 100, and CIS + M 200 groups compared with CTR group and CTR + M 200 groups but it was seen decreased in CIS + M 200 group compared with other groups. When we see the arrangement of the testis tissue we also got the decrease of the germ cells density in the seminiferous tubules in the CIS treated group and the group where 200 mg/kg/day of MOTILIPERM was given just after the CIS is administered in it, and also the disintegration of the Leydig cells that produces the testosterone.

## Conclusions

This study provides sufficient evidence that CIS has a toxic effect on body and reproductive organs by ER stress in the rat. MOTILIPERM decreased these detriments, and so may be potentially valuable in chemotherapy relating infertility.
